# Applications of Antioxidant Secondary Metabolites of *Sargassum* spp.

**DOI:** 10.3390/md21030172

**Published:** 2023-03-09

**Authors:** Marcelo D. Catarino, Rita Silva-Reis, Amina Chouh, Sónia Silva, Susana S. Braga, Artur M. S. Silva, Susana M. Cardoso

**Affiliations:** 1 LAQV-REQUIMTE, Department of Chemistry, University of Aveiro, 3810-193 Aveiro, Portugal; 2 Laboratory of Microbiological Engineering and Application, Department of Biochemistry and Molecular and Cellular Biology, Faculty of Nature and Life Sciences, University of Mentouri Brothers Constantine 1, Constantine 25017, Algeria; 3 Biotechnology Research Center CRBT, Constantine 25016, Algeria

**Keywords:** brown seaweeds, cosmetic, functional foods, phenolic compounds, phlorotannins, phytosterols, terpenoids

## Abstract

*Sargassum* is one of the largest and most diverse genus of brown seaweeds, comprising of around 400 taxonomically accepted species. Many species of this genus have long been a part of human culture with applications as food, feed, and remedies in folk medicine. Apart from their high nutritional value, these seaweeds are also a well-known reservoir of natural antioxidant compounds of great interest, including polyphenols, carotenoids, meroterpenoids, phytosterols, and several others. Such compounds provide a valuable contribution to innovation that can translate, for instance, into the development of new ingredients for preventing product deterioration, particularly in food products, cosmetics or biostimulants to boost crops production and tolerance to abiotic stress. This manuscript revises the chemical composition of *Sargassum* seaweeds, highlighting their antioxidant secondary metabolites, their mechanism of action, and multiple applications in fields, including agriculture, food, and health.

## 1. Introduction

*Sargassum* (family Sargasseae, order Fucales) is a genus of brown algae spanning the ocean basins of the Atlantic, Pacific, and Indian Oceans, inhabiting mostly tropical and subtropical environments where it forms dense submarine forests [1]. In this genus, some species are even holopelagic, and give the name to the Sargasso Sea, bounded only by the currents around the North Atlantic gyre, where species such as *Sargassum fluitans* and *Sargassum natans* create extensive floating mats and a unique ecosystem providing habitats, breeding zones, migration pathways, and feeding areas to diverse marine species [2,3]. On the other hand, due to their invasive nature, *Sargassum* seaweeds may pose a threat to other marine ecosystems. Blooms of these holopelagic species are one of such cases that negatively affect the near-shore seagrass communities, coral colonies, and fauna, ultimately generating socioeconomical problems [2,4]. *Sargassum muticum*, introduced in the north-east Atlantic along the European coasts in the 1970s, is considered one of the most invasive seaweeds in Europe, capable of forming dense beds, replacing native seaweeds [5], and negatively affecting oyster farming and tourism in regions such as Normandy [6]. In turn, in some countries, such as Spain and Portugal, these seaweeds have been considered by some authors as an addition to the algal flora, rather than a threat [7,8], positively impacting the diversity of motile fauna and constituting a potential source of food for fish and cephalopod species [6].

The *Sargassum* genus was first discovered in 1820 and currently comprises near 400 taxonomically accepted species of varying morphologies [1,9]. Morphological plasticity is even observed within the same species in response to environmental conditions, seasons, age and reproductive stage [10]. In general, this genus is characterized by a thallus differentiated into a holdfast, and one to several main axis divided into branches containing leaf-like structures, vesicles, and receptacles (reproductive organ) [1] of diverse shapes and lengths. Excepting *S. natans* and *S. fluitans*, which reproduce only by fragmentation, *Sargassum* seaweeds reproduce sexually [11].

*Sargassum* spp. have been used over the centuries in folk applications [9,10]. For instance, the pharmacological properties of species, such as *S. pallidum*, *S. confusum*, *S. fusiforme*, *S. fullvellum*, *S. henslowianum*, *S. thunbergii*, *S. horneri*, *S. siliquastrum*, *S. muticum*, *S. hemiphyllum*, *S. polycystum,* and *S. vachellianum* are well-recognized in Traditional Chinese Medicine, and their use to treat diseases dates back to nearly 2000 years ago [10]. The most emblematic medicinal application of these seaweeds is, unquestionably, the treatment of thyroid-related disorders such as goiter, mostly due to their high content in iodine. However, *Sargassum* has been used to treat many other pathologies, including scrofula, oedema, arteriosclerosis, skin diseases, and chronic bronchitis, among others [10].

Owing to their composition in nutrients and bioactive compounds, *Sargassum* spp. have high potential for human consumption and animal feed, although some caution is necessary since they can accumulate heavy metals if grown in contaminated environments [2]. In China, *S. fusiforme* is one of the most cultivated and commonly consumed brown algae [12]. In tropical countries, such as the Philippines, *Sargassum* spp. is sometimes used as a cover for preserving fish freshness, and as a vegetable, fertilizer, or even an insect repellent [9]. Concerning animal feed, in the central Philippines, *Sargassum* is usually used for direct consumption or processed into animal feed for exportation [13]. *S. polycystum* and *S. thunbergii* are traditionally used to artificially feed sea cucumber grown in large-scale operations, and other *Sargassum* species, such as *S. horneri,* are being explored as substitutes [14]. Although the literature is still limited, reports on *Sargassum* benefits in animal feed can already be found, showing evidence that *Sargassum wightii* can be used to improve milk yield in Sahiwal cows [15] and *Sargassum* spp. to reduce cholesterol content of shrimp [16]. 

Similar to other seaweeds, *Sargassum* spp. have a long history of application in agriculture as well. In Portugal, “Sargaço” has a secular tradition of being used as a natural fertilizer; in Bermuda, these seaweeds are spread around banana trees as a mulch and fertilizer [17]; in the Caribbean, they are commercialized as mulch, and in Martinique and the Dominican Republic, production of *Sargassum*-based compost is a common practice. 

On top of the above-mentioned applications, due to its richness in bioactive secondary compounds, *Sargassum* biomass is recognized as a valuable resource with high potential for the development of commercially viable products in distinct fields, including healthcare and pharmaceuticals [1]. Among them, phenolic compounds (including phlorotannins), terpenoids, and phytosterols are among the most recognized ones. In seaweeds, they perform vital roles usually working as herbivore deterrents, digestive inhibitors, as antibacterial and antifouling agents, UV protectors among other functions [18], but in the past years, they have been demonstrated to exert numerous bioactive and health promoting activities, specially due to their exceptional antioxidant properties. This work compiles relevant studies that focused on secondary antioxidant metabolites from *Sargassum*, from their basic chemistry to marketed products. 

## 2. Antioxidant Secondary Metabolites 

### 2.1. Phenolic Compounds

Phenolic compounds consist of monomeric, oligomeric, or polymeric compounds characterized by an aromatic ring linked directly to at least one hydroxyl group (–OH). In general, seaweeds are good sources of such compounds, albeit their phenolic profile is extremely variable according to intrinsic and extrinsic factors. In the particular case of this genus, the variations of the phenolic concentrations may range from 1.1% DW to 12.7% DW [19].

**Figure 1:**
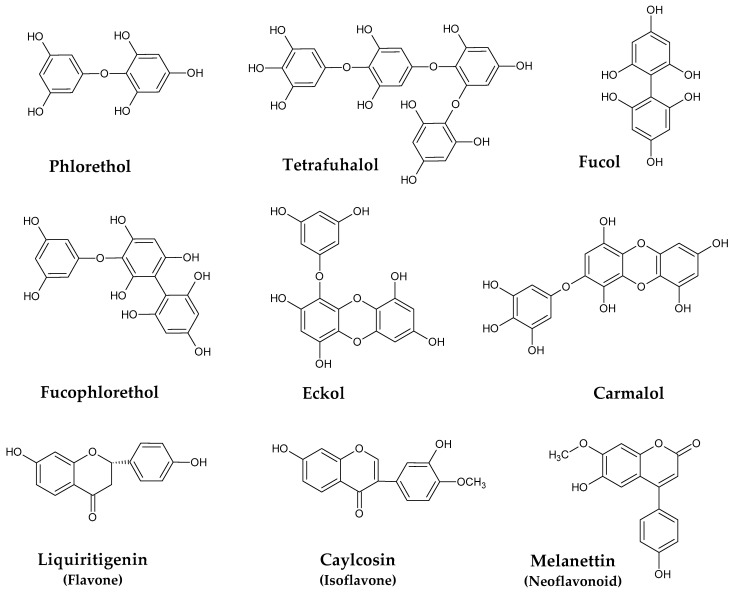
Examples of phenolic compounds isolated from *Sargassum* spp.

Phlorotannins are a characteristic and the most abundant class of polyphenols found in marine brown seaweeds. They are mainly stored in specialized membrane-bound vesicles named physodes [20], and their biosynthesis occurs via the acetate-malonate pathway. There is a large number of phlorotannins structures in nature, with sizes that can range from the simple monomer phloroglucinol, with 126 Da, to very large and complex polymers of 650 kDa [21]. Moreover, variation also occurs according to the type of interphloroglucinol linkage, and within these circumstances, four sub-classes can be pointed out (Figure 1), including phlorethols and fuhalols (ether linkage), fucols (C-C linkage), fucophlorethols (C-C and ether linkages), and eckols and carmalols (dibenzodoxine linkage) [22,23,24]. 

Phlorotannins have gathered much attention during the last few years due to their numerous health properties, which include, among others, antioxidant, anti-inflammatory, and antitumor activities, and even gut microbiota modulatory effects [25,26,27,28,29]. However, the identification and characterization of these compounds is a very complex task due to the large number of similar features, their large size, the high susceptibility to oxidation, and the lack of commercially available standards [30]. Nevertheless, much effort has been made over the years to understand the chemistry of these compounds in distinct algae, including those belonging to the genus *Sargassum*. In general, the levels and the phlorotannins constituents found in seaweeds from this genus depend on factors, such as species, size, age and reproductive status, location, depth, nutrient enrichment, salinity, light exposure, ultraviolet radiation, harvest period, and several others [31]. According to Li et al. [23], fuhalol-type phlorotannins were predominant in a purified fraction of *S. fusiforme* (DP 2–10 monomers), although other relevant compounds ere detected, particularly phlorethols and fucophlorethols, with varying degree of polymerization (DP 2–11 monomers). The same authors also reported newly discovered eckols and carmalol derivatives. Some hydroxyfuhalols (fuhalols with more than one additional hydroxy group), such as hydroxytrifuhalol B, hydroxypentafuhalol A, hydroxyheptafuhalol B, and hydroxynonafuhalol A, were also detected and isolated from *S. spinuligerum* [32], while the most abundant phlorotannins in *S. muticum* collected in Norway were found to belong to fuhalols, hydroxyfuhalols, and phlorethols type [33].

Although less known than phlorotannins, other phenolic constituents in *Sargassum* species have been described as well (Figure 1). In this regard, the isoflavone caylcosin, the flavone liquiritigenin, and distinct neoflavonoids, including melanettin and stevenin, were identified in *S. pallidum* [34]. Other isoflavones, such as daidzin, genistein, sissotrin, formononetin, and biochanin A, were reported to occur in *S. muticum* and *S. vulgare* [35]. 

### 2.2. Terpenoids

Terpenoids represent a large and diverse group of natural occurring products derived from terpenes, i.e., organic compounds consisting of five-carbon isoprene units, containing oxygen molecules. Terpenes or terpenoids are grouped as hemiterpenes (C5), monoterpenes (C10), sesquiterpenes (C15), diterpenes (C20), sesterterpenes (C25), triterpenes (C30), and tetraterpenes (C40).

Carotenoids are the most common tetraterpenes in nature. These have red, yellow, or orange colors, and they are essential for photosynthesis, participating also in photoprotection and in cell membrane stabilization in a wider context. Chemically, carotenoids are divided into carotenes, i.e., pure hydrocarbons containing no oxygen, and xanthophylls, i.e., oxygenated carotenes.

**Figure 2:**
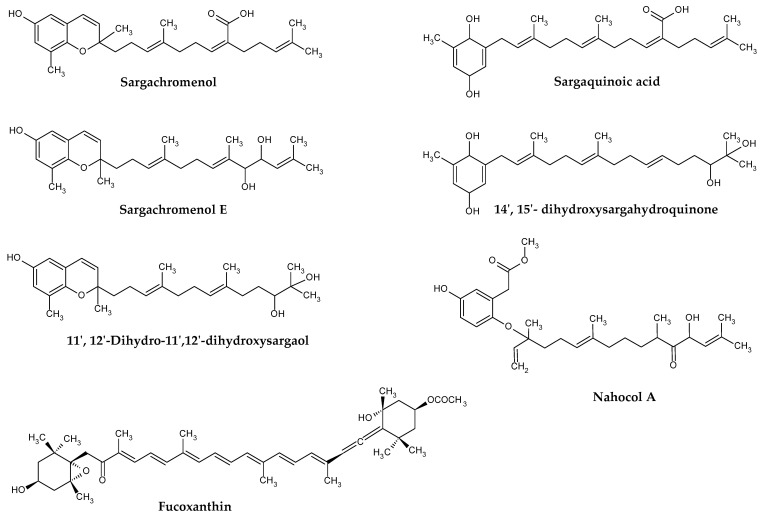
Examples of terpenoids isolated from *Sargassum* spp.

Fucoxanthin (Figure 2) is the major carotenoid produced by brown seaweeds. This orange-colored pigment has a unique chemical structure containing allene bonds, 5,6-monocyclic oxide, and acetylated groups. It occurs in two configurations, designated as *trans* or *cis*, although the former is thermodynamically more stable than the latter [36].

The levels of this pigment are greatly variable between species and depend on the environmental conditions. For instance, in *S. swartzii*, fucoxanthin accounted for 0.17 mg/g dried weight (DW), while concentrations of 0.40 mg/g DW were described in *S. vulgare* and *S. japonica*, and of 0.70 mg/g DW in *S. angustifolium* and *S. plagyophyllum* [37]. These values are much lower than the concentrations of fucoxanthin described for *S. horneri*, *S. fusiforme*, *S. thunbergii*, and *S. confusum* (3.7; 1.1; 1.8; and 1.6 mg/g DW, respectively) from Hakodate, Japan [38]. Moreover, the pattern of seasonal variation is not the same in all species, and the content of fucoxanthin in *Sargassum* varies between species and may be affected by duration of sunshine and seawater temperature. Numura et al. [39] reported concentrations up to 4.49 mg/g DW in *S. horneri* collected in winter, while concentrations of 2.69 mg/g DW were observed in summer.

Brown algae, including those from *Sargassum* genus, also synthesize meroditerpenoids. These compounds have a mixed biosynthetic origin, comprising a polyprenyl chain attached to a *p*-benzoquinone or hydroquinone moiety [29]. The prefix “mero” stands for “part, partial, or fragment”, in Greek terminology, and thus, “meroditerpenoid” are partially derived from diterpenoids. Common precursors of such compounds in algae are geranylgeranyltoluquinol, γ-tocotrienol, and γ-dehydro-tocotrienol.

The first isolated meroterpenoids were γ-tocotrienol, and its 11′,12′-epoxide, from *S. tortile* in 1975 [40]. After that, many meroterpenoids including plastoquinone, chromanol, and chromene have been isolated from different *Sargassum* species (Table 1). Importantly, despite *Sargassum* meroterpenoids exhibit important biological and medicinal properties, no quantifications for these compounds have been reported in the literature so far. 

### 2.3. Phytosterols

Phytosterols are fatty compounds produced by plants, and remarkably contribute as the major lipid constituents of biological membranes of plant cells. *Sargassum* species are considered good sources of phytosterols, such as fucosterol, β-sitosterol, and saringosterol, with low levels of cholesterol. Similar to brown algae in general, fucosterol is the most representative sterol in *Sargassum* seaweeds (see Table 2). Indeed, fucosterol alone was reported to account for 67% of the total phytosterol content in *S. fusiforme* [38]. Ergosterol was also found in relevant concentrations in different species, ranging from 4.0 µg/g DW in *S. piluliferum* to 42.8 µg/g DW in *S. fusiforme*. Moreover, according to Ito et al., seaweeds from the species *S. horneri* collected in different seasons and different locations were found to display significantly different concentrations of phytosterols (12.3–32.3 mg/g DW), demonstrating that these compounds are strongly affected by spatial and seasonal variations [65]. 

## 3. Bioactive Potential of Sargassum Antioxidant Secondary Metabolites 

Oxidation performs a fundamental role in our everyday lives, taking place, for instance, in cell metabolic processes and in food systems. The oxidative metabolism is essential for the survival of cells, but, on the downside, it is responsible of the production of free radicals and other reactive species which, in abnormal conditions, can cause destructive effects by oxidizing membrane lipids, cellular proteins, DNA and enzymes, and even cause disease [70]. In foods, oxidation is the major chemical deterioration, contributing for their rancidification, appearance of off-flavors, unpleasant texture or -color, loss of nutritional quality, and even compromise safety [71]. In this context, natural antioxidants have gathered much interest since they can be used as replacers of the synthetic ones that are used in the food industries and have been linked to multiple health benefits, offering protection against free radicals and oxidative stress in cells, radical-induced tissue injuries, and retardation of the onset and progress of chronic diseases. 

### 3.1. In Chemico Studies

Distinct authors have proven that seaweeds from the genus *Sargassum* have high potential to serve as bio-source of antioxidant compounds. To measure their antioxidant potential, a primary screening is usually carried out using different chemical methods including, among others, radical scavenging activity, protection against lipid peroxidation, metal-ion chelating ability, and reducing capacity, which allow the evaluation of the compounds’ mechanistic intervention, concentration effectiveness, and synergistic effects. A joint summary of the antioxidant activity of *Sargassum* spp. extracts and isolated compounds is compiled in Table 3.

Radical scavenging is one of the most typical mechanisms of antioxidant activity and can be tested via a wide variety of in chemico methods. DPPH and ABTS^+^ radicals are the most commonly used due to their stability, reproducibility, and simplicity. However, although they are useful for an initial screening of the extracts’/compounds’ antioxidant activity, they have low biological relevance since they are synthetic and do not occur in biological systems [68]. For that, radicals, such as NO^•^, O_2_^•−^ or HO^•^, are a more suitable approach since these are biologically produced during normal cellular activities [72]. 

Reducing power is another common method that has been used as a comparative tool among foods and algae. It essentially measures the antioxidant activity of a compound through its ability to stabilize radicals by donating electrons, usually involving the reduction potential of transition metals, such as iron (Ferric Ion Reducing Antioxidant Power, FRAP) or copper (Cupric Reducing Antioxidant Capacity, CUPRAC) [73].

Free radicals can also originate from heavy and transition metals, namely mercury, lead, arsenic, and iron, leading to diseases associated with oxidative stress. Therefore, another mechanism of estimating the antioxidant activity of *Sargassum* compounds is via their capacity to chelate transition metals, forming complex structures which decrease the metal reactivity and eases their excretion from the body.

Other systems, such as β-carotene bleaching or lipid peroxidation, have been reported on compounds extracted from *Sargassum* seaweeds, all of them employed for chemically screen their antioxidant activity of compounds at certain conditions.

Clearly, phenolic compounds, in particular phlorotannins, appear as the major and most well studied group of compounds contributing to the antioxidant properties of *Sargassum* seaweeds. Indeed, positive correlations between phenolic content and antioxidant activity have been reported in many studies using whole algae, their parts, extracts, or fractions [74,75,76,77]. Notably, studies on phlorotannins isolated from *Sargassum* have shown that compounds, such as triphlorethol B, tetraphlorethol C, and pentaphlorethol A, can be more effective than certain antioxidant references such as ascorbic acid or resveratrol at preventing lipid peroxidation or scavenging of O_2_^•−^ radicals [78]. The phenolics group is not, however, the solo contributor for the antioxidant properties of *Sargassum* seaweeds. 

Other molecules, such as pigments have been described for their strong radical scavenging and reducing power properties. This is the case of the pigment-rich extracts retrieved from *S. cristaefolium*, which were shown to exert promising dose-dependent antioxidant properties via DPPH and FRAP assays [79]. Likewise, a positive correlation between the antioxidant activity and the carotenoid content of methanol and ethanol extracts from *S. siliquosum* and *S. polycystum* was described on ABTS^+•^, DPPH^•^ and FRAP [80]. More recently, a carotenoid-rich extract obtained from *S. polycystum* was found to be the most active on DPPH^•^ and ORAC assays compared with those obtained from two other seaweeds, including *Euchema denticulatum* (red) and *Caulerpa lentillifera* (green), most likely due to its high content in fucoxanthin [81]. In fact, within the group of carotenoids, fucoxanthin clearly stands out as one of the most prominent compounds. Due to its structural features, specifically the presence of an allenic bond and a 5,6-monoepoxide motif, fucoxanthin has a strong proton donating capacity that translates into an exceptional antioxidant activity [82]. Indeed, several authors have described strong antioxidant activities in multiple chemical assays (e.g., DPPH^•^, ABTS^+•^, and FRAP) for different *Sargassum* spp.-derived extracts that contained significant concentrations of fucoxanthin [82,83,84]. Moreover, Raji et al. [85] reported that fucoxanthin isolated from *S. wightii* exhibited great radical scavenging properties with IC_50_ values if 79.55 µM and 75.99 µM in DPPH^•^ and ABTS^+•^ assays.

Apart from carotenoids, meroterpenoids have also been described as relevant contributors for the antioxidant properties of the *Sargassum* seaweeds. Among them, sargachromenol (SCM), sargaquinoic acid (SQA), and sargahydroquinoic acid (SHQA) stand out the most, with several authors reporting promising antioxidant activity measured via ABTS^+•^, DPPH^•^, FRAP, OH^•^, and O_2_^•−^ assays for extracts containing these compounds [52,56]. In fact, after isolating SCM and SQA from *S. micracanthum*, Ham and coworkers observed that these two meroterpenoids displayed strong DPPH^•−^ scavenging activities, with the latter being slightly better than the former (49.3 versus 100.2 µM) [86]. Concordantly, the scavenging effects of SCM, SQA, and SHQA isolated from *S. serratifolium* on DPPH^•^ (IC_50_ = 8.0, 15.3 and 5.9 µg/mL, respectively) and OH^•^ (IC_50_ = 0.26, 0.27 and 0.27 µg/mL, respectively) were found promising and even superior to that of the commercial antioxidant reference butyl hydroxytoluene (IC_50_ = 40.4 and 0.9 µg/mL, for DPPH^•^ and OH^•^, respectively) [56]. Likewise, upon isolation from *S. thunbergii*, Seo et al. noticed that these three compounds were as good peroxynitrite (ONOO^-^) scavengers as L-ascorbic acid or penicillamine [87].

Derivatives of these compounds were shown to exert excellent antioxidant properties as well. According to Jang et al., sixteen different sargachromanols isolated from *S. siliquastrum* exhibited significant radical scavenging activity in the range of 87–91% at the concentration of 100 µg/mL [88]. Moreover, mojabanchromanol, isolated from the same species, showed equal or even better results than BHT, L-ascorbic acid, or α-tocopherol on TBARS and DPPH^•^ assays [89]. Identical observations were described for thumbergol A and B, isolated from *S. thunbergii,* which were also as effective as BHT, L-ascorbic acid and α-tocopherol at scavenging DPPH^•^, ONOO^−^, and O_2_^•−^ [60]. In turn, superior antioxidant activities comparing with L-ascorbic acid and α-tocopherol were described for other chromene derivatives retrieved from *S. micracanthum*, including 2-geranylgeranyl-6-methylbenzoquinone and its hydroquinone derivative, performing 28 to 300 times better inhibitory properties on lipid peroxidation than the referred standard compounds [50]. Two other derivatives from 2-geranylgeranyl-6-methyl-1,4-benzohydroquinone isolated from the same species were also found very active against lipid peroxidation, showing an inhibitory effect 40 times stronger than that of α-tocopherol [51].

Apart from these compounds, there are other less studied molecules that can still can contribute importantly for the antioxidant activity of *Sargassum* seaweeds. This is the case of (+)-epiloliolide, which was isolated from *S. naozhouense* and, despite not exerting as good DPPH^•^ scavenging activity as ascorbic acid, it displayed a quite interesting IC_50_ value of 17 mM [54]. In turn, four terpenoids isolated from *S. wightii,* including 2α-hydroxy-(28,29)-*frido*-olean-12(13),21(22)-dien-20-propyl-21-hex-40′(*Z*)-enoate, 2α-hydroxy-(28,29)-*frido*-olean-12(13),21(22)-dien-20-prop-2(*E*)-en-21-butanoate, 2α-hydroxy-8(17),12*E*,14-labdatriene, and 3β,6β,13α-trihydroxy-8(17),12*E*,14-labdatriene exhibited DPPH^•^ and ABTS^+•^ scavenging effects comparable with that of BHT, and significantly better activities in the range of approximately 2–5 times comparing with α-tocopherol [64].

However, one should bear in mind that, for complex samples such as crude extracts, the antioxidant activities are a result of the interactions occurring between multiple compounds that can either lead to synergistic or antagonistic effects. Therefore, the presence of certain compounds individually recognized as antioxidants in a given sample may not translate into the expected antioxidant properties.

**Table 3:** Selected in vitro antioxidant activity tests in non-cellular systems of extracts and purified compounds from *Sargassum* species reported during the last five years.

*Sargassum* spp. Extracts and Compounds				
*Sargassum* spp.	Extraction Conditions	Bioactive Compounds	In Chemico Antioxidant Properties	Ref.
*S. acinarium*	80% MeOH; 80% EtOH; 80% Act; H_2_O	Alkaloids; phenolics, steroids, terpenoids	TAA _MeOH; EtOH; Act; H2O_: 2.1; 2.8; 1.7; 0.7 mg AA/g DW; RP _MeOH; EtOH; Act; H2O_: 0.4; 1.3; 0.4; 0.5 mg AA/g DW	[76]
*S. angustifolium*	EtOH; 50% EtOH; H_2_O	Cardiac glycosides, saponins, steroids, flavonoids	DPPH^●^ _EtOH; 50% EtOH; H2O_: 1/EC_50_ = 14.3; 10.0; 0.7 mg/mL; FICA _EtOH; 50% EtOH; H2O_ (% at 1 mg/mL): 15.6; 19.7; 88.3%; RP _EtOH; 50% EtOH; H2O_ (OD 700 nm at 5 mg/mL): 1.0; 0.4; 0.1; TBARS _EtOH; 50% EtOH; H2O_ (% at 1 mg/mL): 68.9; 23.3; 4.9%	[90]
*S. aquifolium*	EtOH; 50% EtOH; H_2_O	Cardiac glycosides, saponins, steroids, flavonoids	DPPH^●^ _EtOH; 50% EtOH; H2O_: 1/EC_50_ = 2.9; 20.0; 0.7 mg/mL; FICA _EtOH; 50% EtOH; H2O_ (% at 1 mg/mL): 7.3; 20.1; 52.0%; RP _EtOH; 50% EtOH; H2O_ (OD 700 nm at 5 mg/mL): 0.4; 0.5; 0.7; TBARS _EtOH; 50% EtOH; H2O_ (% at 1 mg/mL): 29.3; 10.3; 8.9%	[90]
	EtOH	Phenolics	DPPH^●^: IC_50_ = 828.2 µg/mL	[77]
*S. asperifolium*	EtOH; 50% EtOH; H_2_O	Cardiac glycosides, flavonoids, saponins, steroids, condensed tannins	DPPH^●^ _EtOH; 50% EtOH; H2O_: 1/EC_50_ = 13.3; 40.0; 0.7 mg/mL; FICA _EtOH; 50% EtOH; H2O_ (% at 1 mg/mL): 4.3; 10.9; 42.9%; RP _EtOH; 50% EtOH; H2O_ (OD 700 nm at 5 mg/mL): 1.0; 0.7; 0.2; TBARS _EtOH; 50% EtOH; H2O_ (% at 1 mg/mL): 53.7; 11.3; 3.3%	[90]
*S. boveanum*	EtOH; 50% EtOH; H_2_O	Cardiac glycosides, alkaloids, saponins, steroids, flavonoids	DPPH^●^ _EtOH; 50% EtOH; H2O_: 1/EC_50_ = 100.0; 20.0; 1.2 mg/mL; FICA _EtOH; 50% EtOH; H2O_ (% at 1 mg/mL): 3.9; 23.4; 78.8%; RP _EtOH; 50% EtOH; H2O_ (OD 700 nm at 5 mg/mL): 1.5; 1.1; 0.6; TBARS _EtOH; 50% EtOH; H2O_ (% at 1 mg/mL): 58.9; 20.1; 1.3%	[90]
	Ext: MeOH; Fract: DCM → EtOAc → BuOH → H_2_O	Phenolics	DPPH^●^ _MeOH; DCM; EtOAc; BuOH; H2O_: EC_50_ = 1091.7; 245.9; 171.4; 779.9; 1987.1 ppm; ABTS^●+^ _MeOH; DCM; EtOAc; BuOH; H2O_: EC_50_ = 1204.6; 247.6; 219.5;487.9; 1556.4 ppm; FRAP _MeOH; DCM; EtOAc; BuOH; H2O_: EC_50_ = 535.8; 157.9; 129.2; 243.9; 1381.7 ppm	[91]
*S. cinctum*	H_2_O; MeOH	Phenolics	TAA _H2O; MeOH_: 38.3; 21.9 mg AA/g DW; DPPH^●^_H2O; MeOH_: IC_50_ = 1.1; 1.2 mg/mL; FRAP _H2O; MeOH_: 12.3; 3.7 mg AA/g DW	[91]
*S. coriifolium*	MeOH; EtOH; H_2_O	Terpenoids; saponins; phlorotannins; cardiac glycosides; flavonoids; phenols	DPPH^●^ _MeOH; EtOH; H2O_: IC_50_ = 1.0; 1.4; 2.7 mg/mL; ABTS^●+^ _MeOH; EtOH; H2O_: IC_50_ = 1.6; 2.2; 3.7 mg/mL; H_2_O_2 MeOH; EtOH; H2O_: IC_50_ = 2.0; 2.6; 4.7 mg/mL	[92]
*S. crassifolium*	EtOH	Phenolics	DPPH^●^: IC_50_ = 767.0 µg/mL	[75]
*S. cristaefolium*	EtOH	Phenolics	DPPH^●^: IC_50_ = 737.3 µg/mL	[77]
	Ext: CHCl_3_:MeOH:H_2_O (1:2:0.8) Fract: DCM → Act → MeOH	Fucoxanthin, porphyrin derivatives, galactosyldiacylglycerols	TAA _Ext; DCM; Act; MeOH_: 39.2; 46.2; 66.1; 7.8 ìmol TE/g DPPH^●^ _Ext; DCM; Act; MeOH_ (at 0.5 mg/mL): 41.3; 48.6; 67.2; 11.9% FRAP _Ext; DCM; Act; MeOH_: 688.1; 368.1; 679.2; 132.1 ìmol FE/g	[79]
*S. duplicatum*	EtOH; MeOH; EtOAc	Fucoxanthin	DPPH^●^_EtOH; MeOH; EtOAc_: IC_50_ = 93.8; 78.5; 112.3 ìg/mL	[83]
*S. furcatum*	DCM:MeOH (2:1)	Phenolics	DPPH^●^: EC_50_ = 0.5 mg/L; ABTS^●+^: EC_50_ = 0.3 mg/L	[93]
*S. horneri*	70% MeOH	Phenolics	DPPH^●^: EC_50_ = 06 mg/mL; H_2_O_2_: EC_50_ = 83.9 mg/mL; O_2_^●-^: EC_50_ = 0.5 mg/mL; OH^●^: EC_50_ = 1.4 mg/mL; RP: EC_50_ = 0.2 mg/mL; FICA: 0.4 mg/mL	[94]
	MeOH	Fucoxanthin, phenolics	DPPH^●^ (at 2 mg/mL): 46.5%; RP (OD 700 nm at 2 mg/mL): 0.8	[95]
	Ext: 80% MeOH Fract: CHCl_3_	Phenolics, polysaccharides, sterols, Apo-9 fucoxanthinone	DPPH^●^_Ext; CHCl3_: IC_50_ = 1.1; 2.7 mg/mL; Alkyl	[96]
*S. linearifolium*	80% MeOH → hot H_2_O	Phenolics, alkaloids, terpenoids, sterols	DPPH^●^_hot H2O_: IC_50_ = 124.5 µg/mL; ABTS^●+^_hot H2O_: IC_50_ = 257.1 µg/mL	[97]
	70% EtOH	Fucoxanthin, Phenolics	ABTS^●+^ (at 1 mg/mL): 34.5 mg TE/g extract; DPPH^●^ (at 1 mg/mL): 15.6 mg TE/g DW; FRAP (at 1 mg/mL): 12.5 mg TE/g DW	[84]
*S. miyabei*	70% EtOH	Phenolics	DPPH^●^ (% at 0.1 mg/mL): 42.8%; OH^●^ (% at 0.1 mg/mL): 43.8%; LP (% at 0.1 mg/mL): 52.4%	[98]
	70% EtOH	SHQA, SCM, Phenolics	ABTS^●+^: 186.2 mg VCE/g; DPPH^●^: 193.7 mg VCE/g; FRAP: 1.0 mM FE/g	[52]
*S. muticum*	80% MeOH; 80% EtOH; 80% Act; H_2_O	Alkaloids; phenolics, steroids, terpenoids	TAA _MeOH; EtOH; Act; H2O_: 0.9; 3.1; 2.3; 0.7 mg AA/g DW; RP _MeOH; EtOH; Act; H2O_: 0.2; 1.4; 1.0; 0.7 mg AA/g DW	[76]
	EtOH; Act; EtOAc; CHCl_3_; Hex	Phenolics	DPPH^●^ _EtOH; Act; EtOAc; CHCl3; Hex_: 4.0; 5.4; 16.2; 6.1;4.7 mg TE/g DW; FRAP _EtOH; Act; EtOAc; CHCl3; Hex_: 4.2; 17.5; 18.3; 6.0; 6.2 mg AA/g DW	[99]
	CHCl_3_:MeOH (1:1); SC CO_2_	Glycolipids	DPPH^●^_CHCl3:MeOH; SC CO2_: EC_50_ = 4.1; 0.9 mg/mL	[100]
	CHCl_3_:MeOH (1:1); SC CO_2_	Phospholipids	DPPH^●^_CHCl3:MeOH; SC CO2_: EC_50_ = 4.8; 1.0 mg/mL	[100]
	50% MeOH	Phenolics	DPPH^●^: IC_50_ = 1.64 mg/mL	[101]
	EAE-PLE 4h with alcalase or vicozyme	Phenolics; phlorotannins	TEAC _alcalase; viscozyme_: 0.5; 0.6 mmol TE/g	[102]
*S. naozhouense*	75% EtOH → Column chromatography	(+)-epiloliolide	DPPH^●^: IC_50_ = 17 mM	[54]
*S. oligocystum*	EtOH; 50% EtOH; H_2_O	Cardiac glycosides, alkaloids, saponins, steroids, flavonoids, condensed tannins	DPPH^●^ _EtOH; 50% EtOH; H2O_: 1/EC_50_ = 12.5; 12.5; 5.0 mg/mL; FICA _EtOH; 50% EtOH; H2O_ (% at 1 mg/mL): 7.3; 20.1; 52.0% RP _EtOH; 50% EtOH; H2O_ (OD 700 nm at 5 mg/mL): 1.1; 0.6; 0.5 TBARS _EtOH; 50% EtOH; H2O_ (% at 1bmg/mL): 59.3; 22.0; 2.7%	[54]
*S. podocanthum*	70% EtOH	Fucoxanthin, Phenolics	ABTS^●+^ (at 1 mg/mL): 147.1 mg TE/g extract; DPPH^●^ (at 1 mg/mL): 136.6 mg TE/g DW; FRAP (at 1 mg/mL): 29.6 mg TE/g DW	[84]
*S. polycystum*	CHCl_3_; Hex; MeOH; Act; 70% EtOH	Steroids, phenolics, saponins, terpenoids	TAA _CHCl3; Hex; MeOH; Act; 70% EtOH_ (at 1.25 mg/mL): 68.0; 61.4; 121.0; 46.0; 120.0 mmol AA/g DW	[103]
	EtOH	Phenolics	DPPH^●^: IC_50_ = 804.3 µg/mL	[77]
	H_2_O; MeOH	Phenolics	TAA _H2O; MeOH_: 43.3; 36.1 mg AA/g DW; DPPH^●^_H2O; MeOH_: IC_50_ = 1.0; 1.2 mg/mL; FRAP _H2O; MeOH_: 11.6; 3.0 mg AA/g DW	[104]
	EtOH	Carotenoids	DPPH^●^ (at 1 mg/mL): 20.4%; ORAC: 42.1 mmol TE/100 g	[81]
*S. serrifolium*	70% EtOH	SHQA, SCM, Phenolics	ABTS^●+^: 99.5 mg VCE/g; DPPH^●^: 75.4 mg VCE/g; FRAP: 0.3 mM FE/g	[52]
	70% EtOH → HPLC purification	SHQA, SCM, SQA	ABTS^●+^ _SHQA, SCM, SQA_: IC_50_ = 13.8; 13.1; 47.3 µg/mL; DPPH^●^_SHQA, SCM, SQA_: IC_50_ = 5.9; 8.0; 15.3 µg/mL; O_2_^●-^_SHQA, SCM, SQA_: IC_50_ = 16.9; 14.5; 20.5 µg/mL; ROS: IC_50_ = 0.2; 0.2; 0.3 µg/mL	[56]
*S. tenerrium*	H_2_O; MeOH	Phenolics	TAA _H2O; MeOH_: 52.0; 40.1 mg AA/g DW; DPPH^●^_H2O; MeOH_: IC_50_ = 0.8; 0.8 mg/mL; FRAP _H2O; MeOH_: 13.4; 4.0 mg AA/g DW	[104]
*S. thunbergii*	70% EtOH	Phenolics	DPPH^●^ (% at 0.1 mg/mL): 39.3 %; OH^●^ (% at 0.1 mg/mL): 34.3 %; LP (% at 0.1 mg/mL): 31.0%	[98]
	MeOH	Fucoxanthin, phenolics	DPPH^●^ (at 2 mg/mL): 90.4%; RP (OD 700 nm at 2 mg/mL): 0.5	[95]
*S. vestitum*	70% EtOH	Phenolics	ABTS^●+^: 40.3 mg TE/g DW; DPPH^●^: 111.8 mg TE/g DW; FRAP: 46.2 mg TE/g DW	[105]
	MAE 70% EtOH	Phenolics	ABTS^●+^: 149.8 mg TE/g DW; DPPH^●^: 116.5 mg TE/g DW; FRAP: 68.0 mg TE/g DW	[105]
	UAE 70% EtOH	Phenolics	ABTS^●+^: 147.2 mg TE/g DW; DPPH^●^: 86.1 mg TE/g DW; FRAP: 60.1 mg TE/g DW	[105]
	70% EtOH	Fucoxanthin, Phenolics	ABTS^●+^ (at 1 mg/mL): 183.4 mg TE/g extract; DPPH^●^ (at 1 mg/mL): 209.5 mg TE/g DW; FRAP (at 1 mg/mL): 283.7 mg TE/g DW	[84]
*S. vulgare*	Act	Phenolics	DPPH^●^: IC_50_ = 0.8 mg/mL; O_2_^●-^: IC_50_ = 1.0 mg/mL; RP (OD 700 nm at 1 mg/mL): 0.3	[106]
	MeOH; H_2_O; hot H_2_O	Phenolics	ABTS^●+^ _MeOH; H2O; hot H2O_: EC_50_ = 165.8; 73.3; 46.5 µg/mL; β-CBT _MeOH; H2O; hot H2O_: EC_50_ = 18.2; 142.7; 66.5 µg/mL; FRAP: 216.4; 102.1; 198.3µg TE/mL	[107]
	Ext: 70% Act Fract: Hex → EtOAc → H_2_O	Phlorotannins	ABTS^●+^_Ext; Hex; EtOAc; H2O_: IC_50_ = 72.9; 93.8; 25.1; 74.9 µg/mL; DPPH^●^_Ext; Hex; EtOAc; H2O_: IC_50_ = 97.4; 29.8; 25.8; 96.6 µg/mL O_2_^●-^ _Hex; EtOAc_: IC_50_ = 37.1; 27.0 µg/mL; β-CBT _Ext; Hex; EtOAc_: IC_50_ = 65.2; 41.3; 72.1 µg/mL;	[108]
*S. wightii*	SFE CO_2_:EtOH (94:6); EtOH; 60% EtOH; 40% EtOH; H_2_O	Phenolics, protein	TAA _SFE; EtOH; 60% EtOH; 40% EtOH; H2O_: 67.9; 21.6; 23.0; 31.1; 33.3 mg AA/g extract; FRAP _SFE; EtOH; 60% EtOH; 40% EtOH; H2O_: 57.3; 45.4; 55.7; 62.3; 68.7 mg AA/g extract	[109]
	Hex → EtOAc → MeOH	Phlorotannins	DPPH^●^ _EtOAc_: IC_50_ = 59.9 µg/mL; ABTS^●+^_EtOAc_: IC_50_ = 51.0 µg/mL; FRAP _EtOAc_: 55.2 µg/mL	[110]
	EtOAc → Column chromatography → TLC	Fucoxanthin	DPPH^●^: IC_50_ = 79.6 µM; ABTS^●+^: IC_50_ = 76.0 µM	[85]

Act—Acetone; MeOH—Methanol; EtOH—Ethanol; EtOAc—Ethyl acetate; Hex—Hexane; DCM—Dichloromethane; SFE—Supercritical fluid extraction; MAE—Microwave assisted extraction; UAE—Ultrasound assisted extraction; β-CBT—β-Carotene bleaching test; FICA—Ferrous ion chelating activity; TAA—Total antioxidant activity; RP—Reducing power; LP—Lipid peroxidation; TE—Trolox equivalents; FE: Ferrous equivalent; DW—Dry weight; OD—Optical density, SHQA—Sargahydroquionic acid; SCM—Sargachromenol; SQA—Sargaquionicacid.

### 3.2. Cellular and In Vivo Studies 

Following in chemico screening experiments, the use of biological models is essential to further corroborate the antioxidant activity of a compound and elucidate its mechanisms of action. The screening of the antioxidant activity of *Sargassum* spp. secondary metabolites in cell and animal models has thus been reported by a few authors. In general, oxidative stress inductors, such as H_2_O_2_, carbon tetrachloride (CCl_4_), *tert*-butyl hydroperoxide (*t*-BHP), and 2,2′-azobis(2-amidinopropane) dihydrochloride (AAPH), are applied, while the protective effects of secondary metabolites are estimated through monitorization of oxidative stress markers, such as the levels of intercellular reactive oxygen species (ROS), lipid peroxidation, enzymes, and transcription factors involved in redox homeostasis. Literature data on the antioxidant effects of secondary metabolites from *Sargassum* origin using cellular and in vivo models is summarized in Table 4 and Table 5, respectively. 

#### 3.2.1. Oxidative Stress Protective Effects 

As in chemical studies, distinct authors have emphasized the oxidative stress-protective abilities of *Sargassum* spp. phenolic-rich extracts in biological systems., e.g., Pinteus et al. [111] demonstrated that two purified fractions obtained from the *S. muticum* extract (rich in phenolics, including phlorotannins), namely the MeOH fraction and 50:50 MeOH:DCM fraction, were able to reduce the H_2_O_2_—induced elevation of intracellular ROS in MCF-7 cells by 70% and 56%, respectively. Likewise, phenolic compounds together with sulfated polysaccharides were reported as the major contributors to the antioxidant activity of a *S. polycystum* Celluclast-assisted extract, which was demonstrated to significantly decrease the cell death brought on by H_2_O_2_-induced oxidative stress in zebrafish embryos to normal levels [112]. 

In a different in vivo model, a polyphenol-rich extract of *S. pallidum* containing mainly phlorotannins was reported to decrease lipid peroxidation by 37%, increase the levels of the antioxidant enzyme superoxide dismutase (SOD) levels by 47%, and restore GSH to the control levels in CCl_4_-induced oxidative stress in Wistar rats [113]. Following an identical behavior, a non-identified polyphenol-rich extract of *Sargassum* spp. led to an increase in GPx levels (8.14 ± 4.49 nmol/g) in the liver of seaweed-treated animals when compared to the non-treated CCl_4_-induced Wistar rats (9.88 ± 1.07 nmol/g) [113,114]. Furthermore, after administrating a phlorotannins-rich extract from *S. hemiphyllum* to CCl_4_-exposed Kunming mice, Zhao et al. [115] observed a statistically significant increase in SOD, catalase (CAT), glutathione peroxidase (GPx), and total antioxidant capacity (TAOC) in the serum, kidneys, liver, and brain (*p* < 0.05), which was accompanied by a significant decrease in lipid peroxidation in their livers (*p* < 0.05). 

Terpenoids constitute another important group of compounds of *Sargassum* origin that have shown important antioxidant properties in cell and animal models. Among these, the carotenoid fucoxanthin, isolated from a methanolic extract of *S. siliquastrum*, was proven to significantly decrease ROS production in H_2_O_2_-stimulated Vero cells at 5 µM (*p* < 0.05), 50 µM and 100 µM (*p* < 0.01) [116]. Other terpenoids, such as meroterpenoids, extracted or isolated from several *Sargassum* spp., also demonstrated interesting protective effects on oxidative stress induction. Indeed, after treating *t*-BHP-stimulated HepG2 cells with a meroterpenoid-rich extract of *S. serratifolium* (mainly containing SHQA, SCM, and SQA), Lim et al. [57] showed a statistically significant ROS and lipid peroxidation reduction in a dose-dependent manner (*p* < 0.05). In addition, sargachromanols D, E, and K, and three other chromanols isolated from *S. siliquastrum* at 5 µg/mL, significantly restored GSH in H_2_O_2_-stimulated HT1080 cells [59]. 

A similar trend was described for (−)-loliolide, a different type of monoterpenoid, which was able to significantly reduce ROS levels (*p* < 0.01) on AAPH-stimulated Vero cells and zebrafish embryos at concentrations of 12.5 and 25 µg/mL. Interesting inhibition of lipid peroxidation was observed in a different study using zebrafish embryos as well [44]. Notably, an indole derivative, namely indole-6-carboxaldehyde, isolated from *S. thunbergii*, was found interfere strongly with the cellular antioxidant defenses of V79-4 cells, triggering a significant increase in the expression heme oxygenase-1 (HO-1), and most importantly, in nuclear factor-erythroid 2-related factor 2 (Nrf2), an important transcription factor that regulates the expression of intracellular antioxidant proteins [62].

#### 3.2.2. Effects on Oxidative-Stress Related Disorders

Multiple diseases are frequently linked to oxidative stress. Considering this, many in vitro and in vivo experiments of *Sargassum* spp. revealed a variety of protective properties against diseases linked to the antioxidant activity associated with the secondary metabolites present in this brown seaweed. 

UV irradiation leads to increased inflammation and skin damage through the induction of ROS. In UV-B irradiated HaCaT cells, i.e., immortalized aneuploid keratinocytes from adult human skin, extracts and/or compounds from different *Sargassum* species have been shown to decrease ROS production, lipid peroxidation, and increase antioxidant defense through induction of SOD and CAT activity. Accordingly, Piao et al. [117] and Han et al. [118] demonstrated that ethanolic and methanolic extracts from *S. muticum* and *S. horneri*, respectively, triggered a significant increase in SOD and CAT activity (*p* < 0.05), and a decrease in ROS and lipid peroxidation in UV-B-exposed HaCaT cells at all tested concentrations (*S. muticum*—12.5 µg/mL, 25 µg/mL, 50 µg/mL, 100 µg/mL, and *S. horneri*—31.6 µg/mL, 62.5 µg/mL, and 125 μg/mL, respectively). The authors also found a good correlation between their results observed and the high percentage of polyphenols commonly present in the extracts. *S. horneri* extract contained 7.45 ± 0.29% of total phenolics and 0.29 ± 0.07% of total flavonoids. In a different model using UV-irradiated zebrafish embryos, a polyphenol-rich ethanolic extract from *S. thunbergii*, demonstrated a decrease (at 0.8 µg/mL, 1.2 µg/mL, and 1.6 μg/mL concentrations, respectively) in ROS production by 71%, 71%, and 85%, respectively [119]. Fucoxanthin and the meroterpenoid tetraprenyltoluquinol chromane, isolated from *S. siliquastrum* and *S. muticum,* respectively, showed a similar trend in human dermal fibroblasts exposed to the same radiation [53,120]. Other terpenoids, such as (−)-loliolide and sargachromanol (SC) E, isolated from *S. horneri*, caused a reduction in ROS production induced by UV irradiation, in HaCaT cells and human dermal fibroblasts, respectively. Thus, (−)-loliolide only caused a significant inhibition at 12.5 µg/mL and 25 μg/mL, while SC E in all concentrations tested (5 µg/mL, 10 µg/mL, and 20 µmol/L) [45,47].

The effects of two *S. virgatum* extracts with high contents of polyphenols on spermatogenesis and infertility of male Wistar rats exposed to γ-irradiation were shown to increase the antioxidant enzymes SOD, CAT, GPx, and GSH at 100 mg/kg body weight (*p* < 0.05). At 400 mg/kg, the ethanolic extract displayed the best effect in the increased antioxidant enzymes: 86.38% for SOD, 80.87% for GPx, 90.04% for CAT, and 81.55% for GSH compared with the controls [121]. In a different approach, fucoxanthin, isolated from *S. glaucescens*, also stimulated these enzymes’ activity in plasma and testicles from Syrian hamsters, which were lowered by cisplatin exposure, a chemotherapeutic agent that is usually associated with infertility [122]. 

Accumulation of particulate matter in the lungs is another common cause that triggers oxidative stress and can lead to serious tissue damage and cardiopulmonary complications [123]. In this context, the treatment of particulate matter-exposed HaCaT and murine lung epithelial cells with polyphenol-rich *S. horneri* ethanol extracts exhibited promising effects towards counteracting ROS generation and lipid peroxidation, while notably improving SOD and CAT activities at 62.5 µg/mL and 125 µg/mL [123,124]. Similarly, in particulate matter exposed HaCaT cells and zebrafish embryos, a fucoxanthin-rich extract of *S. fusiformis* caused a reduction in several factors associated with inflammatory responses, including ROS production, reaching statistical significance at 25 µg/mL, 50 µg/mL, and 100 µg/mL [125]. Other compounds, such as (–)-loliolide, isolated from *S. horneri*, or fucosterol isolated from S. *binderi*, demonstrated interesting activities as well. *S. horneri* revealed a great capacity to boost cell viability through ROS reduction in fine dust-exposed HaCaT cells [46], while *S. binderi* not only caused a dose-dependent (12.5 µg/mL, 25 µg/mL, 50 µg/mL, and 100 µg/mL) increase in SOD and CAT activity, but also triggered a significant increase in HO-1 levels and Nrf2 nuclear translocation [126]. 

The synthetic drug methamphetamine (MA) has a neurodegenerative effect on the human brain and can increase the generation of ROS through dopamine oxidation. In fact, a hydroethanolic extract of *S. angustifolium* rich in polyphenols, including gallic acid, protocatechuic acid, gentisic acid, and hydroxybenzoic acid, led to a decrease in ROS generation on MA-exposed SH-SY5Y dopaminergic cells with doses of 80 and 160 μg/mL [127]. Similarly, fucoxanthin isolated from *S. horneri* prevented the effects of MA on ROS, significantly reducing their production (*p* < 0.05) and causing a significant increase in HO-1 expression in PC12 cells at 3 µM [128].

Researchers frequently induce inflammatory reactions in RAW 264.7 cells by exposing them to lipopolysaccharide (LPS). It can stimulate membrane-bound NADP oxidase and consequently, the generation of ROS. Ethanol, methanol, and ethyl acetate *S. serratifolium* extracts, whose major compounds identified were the meroterpenoids SHQA, SC, and SQA, were reported to dose-dependently (1.25–10 µg/mL) reduce LPS-induced ROS in LPS-stimulated macrophages [56]. The same trend was observed with SCM and SHQA isolated from *S. horneri* and *S. macrocarpum*, respectively [48,49]. In turn, an acetone:ethanol extract of *S. glaucescens* rich in fucoxanthin caused a decrease in LPS-induced ROS and O_2_^•−^ production in the same cell line at concentrations ranging from 25 µg/mL to 100 µg/mL [122]. The anti-inflammatory properties of fucoxanthin are further evidenced by in vivo studies in rodents [129,130], including scenarios of induced asthma [131] and induced colon inflammation [132]. 

ROS are produced in high quantity in the mitochondria in scenarios of hyperglycemia and in the case of other metabolic disorders such as diabetes. In this sense, Lee et al. [133] treated glucose-stimulated INS-1 cells with an extract of *S. sagamianum* with the extract at 100 µg/mL and observed a reduction of ROS (by 122%) and lipid peroxidation, attributing these effects to the presence of secondary metabolites, such as phlorotannins and plastoquinones. A hydroethanolic extract of *S. fusiformis* rich in several secondary metabolites such as flavonoids, fucoxanthin, and fucosterol, also afforded a statistically significant increase in SOD and CAT activities accompanied by a notable decrease in lipid peroxidation in a high-fat diet and streptozotocin-induced ICR mice [134]. In fact, studies using diabetic mice showed that fucoxanthin lowers fasting glucose levels, increases plasma insulin levels and, when administered in tandem with metformin, induces the regeneration of pancreatic β-cells [135,136]. Usually diabetic mice have altered serum lipid profiles, with elevated values of triglycerides and cholesterol, which are reduced when treated with fucoxanthin [135]. These anti-diabetic and lipid profile regulating properties of fucoxanthin are the target of a clinical trial in patients with metabolic syndrome, currently under phase II [137].

Three phlorotannins isolated from *S. carpophyllum* were identified as anti-allergic agents. The compounds inhibited the activation of mast cells, the cells of the immune system responsible for the release of histamine, particularly when triggered by an allergen [138]. In addition to inhibiting the formation of ROS, phlorotannins are promising candidates for novel anti-allergic drugs. Choi et al. [54] showed that SHQA, isolated from *S. serratifolium*, prevented the activation of effector cells in allergic responses and caused a statistically significant reduction in ROS formation, once again demonstrating the antioxidant properties of meroterpenoids present in *Sargassum* spp.

In addition to the previously reported disease-counteracting effects, *S. angustifolium* hydroethanolic extracts rich in phenolic compounds were shown to dose-dependently (at 20 mg/kg, 40 mg/kg, and 80 mg/kg) improve defense against oxidative stress in rats, through the notable increase in the total antioxidant capacity in rats’ blood, and the decrease in lipid peroxidation brought on by hypertension and dyslipidemia, respectively [139,140].

**Table 4:** Antioxidant effects of *Sargassum* spp. Secondary metabolites in cellular models.

*Sargassum* spp.	Solvents	Secondary Antioxidants	Cells	Oxidative Stress Inductors	Effects	Ref.
*S. angustifolium*	80% EtOH	PPS	SH-SY5Y	Methamphetamine	↓ ROS	[127]
*S. fusiformis*	Ext: 80% MeOH Frac: Chl and water	fucoxanthin (↑%)	HaCaT	Particulate matter exposure	↓ ROS	[125]
*S. glaucescens*	Ext: 70% Ace in EtOH Frac: 90% EtOH, EtOH:water:Hex (9:1:10)	fucoxanthin (↑%)	RAW 264.7	LPS-stimulation	↓ ROS/O_2_^-^	[122]
*S. horneri*	80% MeOH	PPS (↑%)	HaCaT	UV-B	↓ ROS	[118]
	95% EtOH	PPS (↑%)	MLE-12	Particulate matter	↓ ROS/LPO; ↑ SOD/CAT	[123]
	70% EtOH	PPS (↑%),	HaCaT	Fine dust	↓ ROS	[124]
	Ext: 70% EtOH Frac: Hex, DCM, EtOAc, BuOH	Fucosterol	BV2 HT22	LPS (BV2 cells); Glu (HT22 cells)	BV2 cells: ↑ HO-1/Nrf2 (DCM fraction) HT22 cells: ↓ ROS (DCM fraction)	[141]
*S. muticum*	Ext: MeOH:DCM Frac: MeOH:DCM, MeOH	PPS	MCF-7	H_2_O_2_	↓ H_2_O_2_	[111]
	Ext: 80% EtOH Frac: Hex, DCM, EtOAc, BuOH, H_2_O	PPS	HaCaT	UV-B	↓ ROS/LPO; ↑ SOD/CAT	[117]
*S. plagiophyllum*	Distilled water	PPS	Human normal colon	H_2_O_2_	↓ ROS	[142]
*S. polycystum* and *S. natans*	Enzymes: Viscozyme, Celluclast, AMG, Termamyl, and Ultraflo	PPS	Chang	Extraction with different enzymes	↑ ROS scavenging effects in Celluclast Ext	[112]
*S. sagamanum*	80% EtOH	PPS	INS-1	Glucose	↓ ROS/LPO	[133]
*S. serratiflium*	70% EtOH	SHQA, SC, SQA (↑%)	HepG2	t-BHP	↓ ROS/ LPO; Prevention of GSH oxidation; ↓ SOD/CAT; ↑ GST; ↑ Nrf2 Independent of t-BHP: ↑ HO-1/Nrf2	[57]
	Ext: EtOAc, MeOH, EtOH, Ace, Hex, Chl, H_2_O	SHQA, SC, SQA (↑%)	RAW 264.7	LPS	↑ TPC (EtOAc, MeOH and EtOH Ext) ↓ ROS (EtOAc, MeOH, and EtOH Ext)	[56]
*S. thunbergii*	40% EthOH	PPS (↑%)	L929	UV-B	↓ ROS/LPO; ↑ SOD and CAT	[119]
*S. binderi*	Ext: 70% EtOH Frac: Hex, Chl, EtOAc	Fucosterol	A549	Fine dust	↓ ROS; ↑ HO-1, SOD, CAT, and Nrf2	[126]
*S. carpophyllum*	Ext: 80% MeOH Frac: Chl and H_2_O	3 phlorotannins	RBL-2H3	DNP-HAS	↓ ROS	[138]
*S. horneri*	Ext: 80% MeOH Frac: Chl	(−)-Loliolide	Vero	AAPH	↓ ROS	[44]
	Ext: 80% MeOH Frac: Hex, EtOAc, MeOH, H_2_O	(−)-loliolide	HaCaT	UV-B	↓ ROS	[45]
	Ext: MeOH Frac: Hex, 85% Aq MeOH, BuOH, H_2_O	SC E	HDF	UV-A	↓ ROS, LPO and membrane protein oxidation	[47]
	Ext: 80% MeOH Frac: Hex, Chl, EtOAc	(−)-loliolide	HaCaT	Fine dust	↓ ROS	[46]
	EtOH	Fucoxanthin	PC12	Methamphetamine	↓ ROS; ↑ SOD and CAT; ↑ HO-1 and Nrf2	[128]
	Ext: 70% EtOH Frac: Hex, EtOAc	SCM	RAW 264.7	LPS	↓ ROS; ↑ HO-1/Nrf2	[48]
	Ext: 70% EtOH Frac: Hex	Fucosterol	HDF	TNF-á/IFN-ã	↓ ROS; ↑ HO-1/Nrf2	[143]
*S. macrocarpum*	80% EtOH	SHQA	RAW 264.7	LPS	↓ ROS; ↑ HO-1	[49]
*S. muticum*	Ext: MeOH Frac: Hex, DCM, EtOAc, BuOH	TPM	HDF	UV-A	↓ ROS	[53]
*S. serratifolium*	Ext: 95% EtOH, H_2_O/ EtOH Frac: Hex, EtOAc, BuOH, H_2_O	SHQA	KU812F	PMACI	↓ ROS	[144]
*S. siliquastrum*	Ext: 80% MeOH Frac: Chl	Fucoxanthin	Vero	H_2_O_2_	↓ ROS	[116]
	Ext: MeOH Frac: Hex, 85% Aq MeOH, BuOH, H_2_O	SCM D, E, and K, 3 chromanols	HT1080	H_2_O_2_	↓ ROS/LPO; ↑ GSH	[59]
	Ext: 80% MeOH Frac: Chl	Fucoxanthin	Human fibroblasts	UV-B	↓ ROS	[120]
*S. thunbergii*	Ext: 80% MeOH; Frac: Chl	I6CA	V79-4	H_2_O_2_	↓ ROS; ↑ HO-1 and Nrf2	[62,63]
	Ext: MeOH Frac: Hex, 85% aq MeOH, BuOH, H_2_O	SC E, SC D, SHQA	HT1080	Fe(II)/H_2_O_2_; AAPH	↓ ROS; ↓ LPO	[61]

↓: decreased; ↑: increased; AAPH: 2,2′-azobis(2-amidinopropane)dihydrochloride; Ace: acetone; Aq: aqueous; BuOH: butanol; CAT: catalase; Chl: chloroform; CHO: carbohydrates; CMF-DA: 5-chloromethylfluorescein diacetate; DCM: dichloromethane; DCFH-DA: 2′,7′-dichlorodihydrofluorescein diacetate; DPPP: diphenyl-1-pyrenylphosphin; DNP-HAS: dinitrophenyl-human serum albumin; EtOAc: ethyl acetate; EtOH: ethanol; Ext: extract; Frac: fractionation; Glu: glutamate; GSH: reduced glutathione; GST: glutathione S-transferase; H_2_O: water; H_2_O_2_: hydrogen peroxide; HDF: human dermal fibroblasts; Hex: Hexane; HO-1: heme oxygenase-1; HX: cyclohexane; I6CA: Indole-6-Carboxaldehyde; LPO: lipid peroxidation; LPS: lipopolysaccharide; mBrB: monobromobimane; MDA: malondialdehyde; MeOH: methanol; NBT: nitro blue tetrazolium; ND: non-defined; Nrf2: nuclear factor-erythroid 2-related factor 2; O^2−^: anion superoxide; PMACI: phorbol.

**Table 5:** Antioxidant effects of *Sargassum* spp. Secondary metabolites in vivo models.

*Sargassum* spp.	Solvents	Secondary Antioxidants	Animal Model	Oxidative Stress Inductors	Effects	Ref.
ND	H_2_O	PPS (↑%)	Wistar rats	CCl_4_	↑ GPx	[114]
*S. angustifolium*	70% EtOH	PPS (↑%)	Wistar rats	Dexamethasone	↓ LPO	[140]
	70% EtOH	PPS	Wistar rats	CdCl_2_	↑ TAOC	[139]
*S. fusiformis*	Ext: 80% MeOH Frac: Chl, H_2_O	fucoxanthin (↑%)	Zebrafish embryos	Particulate matter	↓ ROS	[125]
	95% EtOH	Flv, fucoxanthin, fucosterol	ICR mice	HFD/STZ	↓ LPO; ↑ SOD and CAT	[134]
*S. glaucescens*	Ext: 70% Ace in EtOH Frac: 90% EtOH, EtOH: H_2_O:Hex	Fucoxanthin (↑%)	Syrian hamsters	Cisplatin chemotherapy	↑ SOD (plasma); ↑ GPx and CAT (testicles)	[122]
*S. hemiphyllum*	Ext: 70% EtOH Frac: EtOAc	Phlorotannins	Kunming mice	CCl_4_	↑ SOD (serum, kidney); ↑ TAOC (serum, kidney, and liver); ↑ CAT (kidney, brain, and liver); ↑ GPx (brain and liver); ↓ LPO (kidney)	[115]
*S. longifollium*	EtOH	Terpenoids, PPS, and Flv (encapsulated in Na-Caseinate matrix)	Fingerling Tilapia	*A. salmonicida*	SOD and LPO levels returned to normal	[145]
*S. pallidum*	70% aq Ace	PPS (↑%)	Wistar rats	CCl_4_	↓ LPO; ↑ SOD and GSH	[113]
*S. policystum*	Enzymes: Celluclast	PPS	Zebrafish embryos	H_2_O_2_	↓ ROS	[112]
	H_2_O	PPS (↑%)	Sprague–Dawley rats	HCF diet	↓ LPO; ↑ SOD	[146]
*S. thunbergii*	Ext: 40% EtOH Frac: EtOAc	PPS (↑%)	Zebrafish embryos	UV-B	↓ ROS	[119]
*S. virgatum*	EtOH	PPS	Wistar rats	ã-irradiation	↑ SOD, CAT, GPx, and GSH	[121]
*S. vulgare*	MeOH	↑ Total phenolics and Flv	Wistar Rats	ASP	↓ LPO; ↑ SOD and CAT	[147]
*S. horneri*	Ext: 80% MeOH Frac: Chl	(−)-Loliolide	Zebrafish	AAPH	↓ LPO and ROS	[44]
	Ext: 80% MeOH Frac: Hex, EtOAc, MeOH, H_2_O	(−)-Loliolide	Zebrafish	UV-B	↓ LPO an ROS	[45]

↓: decreased; ↑: increased; AAPH: 2,2′-azobis(2-amidinopropane)dihydrochloride; Aq: aqueous; CAR: carotene; CAT: catalase; CCl4: carbon tetrachloride; CdCl_2_: cadmium chloride; Chl: chloroform; CHO: carbohydrates; DCFH-DA: 2′,7′-dichlorodihydrofluorescein diacetate; DEN: diethylnitrosamine; DPPP: diphenyl-1-pyrenylphosphin; EtOH: ethanol; EtOAc: ethyl acetate; Ext: extract; Flv: flavonoids; Frac: fractionation; GPx: glutathione peroxidase; GSH: reduced glutathione; H_2_O: water; H_2_O_2_: hydrogen peroxide; HCF: high cholesterol/high fat; Hex: hexane; HLP: hyperlipidemia; HSD: high stocking density; LPO: lipid peroxidation; LPS: lipopolysaccharide; MDA: malondialdehyde; MeOH: methanol; MNNG: *N*-methyl-*N*’-nitro-*N*-nitrosoguanidine; NaCl: sodium chloride; ND: non defined; PFP: sodium pentafluoropropionate; PPS: polyphenols; RBC: red blood cells; ROS: reactive oxygen species; SA: S. aquifolium; SHE: *S. horneri* extract; SOD: superoxide dismutase; STZ: streptozotocin; TAOC: total antioxidant capacity; TCP: tocopherol; Vit C: vitamin C.

## 4. Applications in Non-Patented and in Patented Products

### 4.1. Food Applications

Apart from the well-known Asian cultural uses of *Sargassum* seaweeds as a food ingredient, these marine resources have recently raised much interest in applications in novel food compositions. Indeed, we have recently witnessed the appearance of products, such as a health-promoting tea, in which powdered *Sargassum* (3.3%) is combined with green tea, other seaweeds, and herbs [148], and a cocktail developed in the Caribbean called ‘pineapple gift’, which contains pineapple as a base, *Sargassum* extract, lemon juice, aquafaba, lavender, angostura bitters, and tequila [149]. Moreover, the incorporation of *S. marginatum* in pasta has been shown to improve its antioxidant properties and decrease pasta strands stickiness after cooking [150].

In South Korea, the development of innovative food formulations containing antioxidant-rich extracts of *Sargassum* is a growing practice, with focus on baked products, including bread and cakes. *S. sagamianum* antioxidant-rich extracts were incorporated into bread at doses of 0.25%, 0.50%, and 0.75%, and evaluated regarding antifungal and sensorial properties. The extracts, at a dose of 0.75%, were able to keep the bread free from mold for up to six days and significantly reduced the mold infestation in bread after nine days of storage when compared to the control (0% seaweed extract), thus translating into an increased shelf-life. The color of the enriched bread was darker compared to the control, with reddish-brown tones. The change in color, along with bitter taste and seaweed smell, made the enriched bread less accepted by panelists, following negative correlation between seaweed concentration and panelists’ acceptance [151]. Other novel seaweed-containing breads have been reported, including bread enriched with an extract of *S. fulvellum* [152] or an ethanol extract of *S. siliquastrum* [153]. The fortified breads, at doses of 1% and 2% in the case of *S. fulvellum* extract, and 0.1% and 1% in the case of *S. siliquastrum* extract, presented a slightly higher resistance against oxidation of the lipids in their composition and bacterial colonization with *Bacillus subtillis*. However, the extracts did not seem to reduce the ability of molds to grow. The higher doses of extracts caused breads to present unpleasant color, taste and aroma that made them less preferred by the sensorial evaluation panel. Nevertheless, the bread with 0.1% *S. siliquastrum* extract allowed combining increased resistance to microorganisms without a significant alteration in the overall sensorial properties.

*S. fusiforme* residue containing antioxidants and fiber was used as a fortifying ingredient in sponge cake. The residue was prepared by removing polysaccharides from *S. fusiforme* (with hot water), drying and superfine grinding to c.a. 10 μm particles. Sponge cake with *S. fusiforme* powder at a ratio of 10% presented a significant difference to control in color analysis, affording increased redness. It was, however, well rated on sensorial analysis, with slightly higher overall preference scores than the control sponge cake [154]. Combining an attractive orange color with antioxidant and nutraceutical properties, fucoxanthin is a useful ingredient for the food processing industry. In Japan, *Sargassum* is expected to become a most relevant source of food-grade fucoxanthin, as the preparation and subsequent incorporation of fucoxanthin-rich extracts from several species of *Sargassum* is the subject of a pending patent. The sources of fucoxanthin listed in this patent include *S. fusiforme, S. sagamianum, S. yezoense, S. yendoi, S. horneri, S. thunbergii, S. patens, S. confusum,* and algae from other genera [155]. In China, fucoxanthin-fortified meat products are patented for ‘improving DHA (docosahexaenoic acid) level in a human body’ [156]. Incorporation of fucoxanthin, obtained from *Sargassum* sp., into catfish sausages at 1%, 2%, 3%, or 4% mass percentage intensifies the color of the product and slightly increases its stability against microbial degradation [157]. The hedonic test of catfish sausages with the different concentrations of fucoxanthin shows significant differences (*p* < 0.05) for appearance, odor, flavor, texture, color, and overall quality score, with the catfish sausage with 1% fucoxanthin being the most preferred by the panelists.

### 4.2. Cosmetic Applications

Asian countries have also a long tradition of incorporating *Sargassum*, either as a whole plant or in the form of extracts, into cosmetic and skin-regenerating formulations [158]. Several aspects need to be considered for these applications to be feasible, which include the extraction yields of the target compounds, the amounts necessary to have a desirable effect, and the stability of the compounds in a certain formulation, among others. Nevertheless, there are currently various patented facial masks based on whole seaweeds of *Sargassum* aimed at skin hydration, nourishing and anti-aging, even though the active components responsible for the skin improvement effect are not identified [159,160,161,162,163,164]. Cosmetics based on extracts of *S. muticum* and *S. serratifollium* have also been patented for skin improvement, with anti-cellulite, skin-lightening, and/or anti-wrinkle benefits [165,166].

The broad range of cosmetic properties described for *Sargassum* in the abovementioned patents is related to the diversity of active components present in this seaweed, including phloroglucinol, phenolic compounds and/or phlorotannins, and fucoxanthin.

Phloroglucinol is used in a variety of cosmetics, mostly as a skin renewal and hair dyeing agent. In fact, it was patented by L’Oreal as an epidermal renewal-promoting agent under the claim of inducing keratinocyte proliferation [167]. With the expiry of the patent in 2018, it became available for any company to include in their compositions. Currently, phloroglucinol (in the trimethyl ether form) is found in various skincare formulations, including anti-wrinkle lotions, night creams, and lifting serums [168]. The application of phloroglucinol as a hair colorant is based on its ability to generate red color upon reaction with compounds, such as aldehydes or allyl groups [169]. Phloroglucinol can be found in several oxidative hair dye formulations, at concentrations ranging between 0.1% and 1% [170].

Polyphenolic extracts have properties that make them interesting ingredients for cosmetics formulation. These are typically dose-dependent, that is, they are more potent as the total phenolic content in the extract increases. For instance, the antimicrobial properties of the polyphenols from *S. polycystum* are dose-dependent, and they make this extract an effective preservative agent to replace chemical additives in lotions [171].

In France, antioxidant-rich extracts of *S. muticum* have been patented for use in cosmetics with the purpose of protecting against reactive oxygen radicals and maintaining a young skin appearance; the patent has expired in April 2022 [172]. In South Korea, polyphenol-rich extracts of a mixture of *Sargassum horneri* and *Enteromorpha prolifera* are patented as antioxidants for use in cosmetic formulations [173]. The polyphenols in *S. plagyophyllum* have, in addition to the antioxidant action, the ability to inhibit collagenase, making them useful for the preparation of natural anti-wrinkle products [174].

The ability to inhibit tyrosinases is another characteristic with cosmetic interest observed in *Sargassum* phlorotannins [175]. This class of enzymes is involved in the production of the skin pigment, melanin, and compounds able to inhibit them are much sought after for their skin whitening effect. Moreover, phlorotannins are trending ingredients in the industry of natural cosmetics because they can combine anti-aging and UV-protecting activities. A phlorotannin-rich fermented extract of *S. vulgare*, registered under the tradename DermalRx^®^ FSE, is available as a natural ingredient for cosmetics from the biotechnological company Biocogent. The product is claimed to provide ‘strong anti-pollution activity’ while inhibiting oxidation and inflammation, being indicated by the manufacturer as most suited for incorporation into cosmetic products with protective and repairing properties [176].

Fucoxanthin, a carotenoid with important radical-scavenging and anti-UV properties, is employed in a variety of cosmetics, from sun lotions and firming serums to more specialized formulas that treat acne and help accelerate scar regeneration [177]. Moreover, its ability to interfere with the metabolism of lipids is making this compound an attractive ingredient for slimming cosmetic formulations. An anti-cellulite cosmetic composition based on fucoxanthin (e.g., from *S. fulvellum*) was patented under the claim of inducing apoptosis of the fat cells, thereby reducing cellulite [178].

### 4.3. Applications as Biostimulants

Although there is a long tradition of applications of *Sargassum* as fertilizers in agriculture, the effects of this genus as biostimulants are still not well explored. Nevertheless, some there are already some reports describing promising applications of these seaweeds as biostimulants. Notably, Sembera et al. [179] reported a compost based on *Sargassum* with equal or superior quality to current compost standards. Moreover, over the last years, *Sargassum* has been explored regarding its potential to be used as crop biostimulants and/or novel products for crop protection. In this regard, [180] demonstrated the positive effect of *S. angustifolium* extracts on the growth of *Lens esculenta*. Additionally, a *Sargassum* spp. compost was described to improve mangrove seedlings development in dry nurseries, a fact that can represent a promising strategy in mangrove restauration [31]. *S. vulgare* extracts were also reported to increase the germination of *Triticum durum* [181] and *Phaseolus vulgaris* [130] under salt stress. Regarding crop protection, Shahriari and coworkers [129] recently highlighted the potential of *S. angustifolium* extracts to the improvement of drought tolerance in *Brassica napus* L., while Han et al. [131] remarked the positive effect of *S. horneri* extracts on the thermal tolerance of *Neopyropia yezoensis* algae. In a different approach, the treatment of *Solanum lycopersicum* with *S. tenerrimum* and *S. fusiforme* extracts was found to enhance its resistance to the fungi *Macrophomina phaseolina,* [132] and to reduce the late blight and gray mold diseases [182], respectively.

## 5. Concluding Remarks

A variety of antioxidant compounds found in *Sargassum* seaweeds have been proven capable of improving shelf-life, nutritional, and public health status as food, food adjuvants, and pharmaceutical or phytopharmaceutical/biostimulant products. Currently, there are new potential markets for such seaweeds and growing interest on the exploitation of their products as natural sources with high antioxidant activity. This trend is clearly expressed by the increasing amount of research and innovation that has been taken in this field during the recent years with great prominence in the food and cosmetic sectors which are putting strong efforts in the development of new products containing extracts and/or antioxidant compounds from this genus. Nevertheless, joint efforts of research and industries are still necessary in order to raise awareness to the full potential of these natural marine resources and build an economical value chain.

## Figures and Tables

**Table 1:** Reported meroterpenoids in *Sargassum* spp.

Species	Meroterpenoids	Ref.
*S. autumnale*	Nahocol (Isomers: A; A1; B; C; D1; D2) Isonahocol (Isomers D1; D2)	[41]
*S. fallax*	Fallahydroquinone Fallaquinone Fallachromenoic acid	[42]
*S. horneri*	Mojabanchromanol	[43]
	(−)-Loliolide	[44,45,46]
	Sargachromanol E	[47]
	Sargachromenol	[48]
*S. macrcarpum*	Sargahydroquinoic acid	[49]
*S. micracathum*	2-geranylgeranyl-6-methylbenzoquinone	[50]
	2-geranylgeranyl-6-methyl-1,4-benzohydroquinone	[51]
*S. miyabei*	Sargahydroquinoic acid Sargachromanol	[52]
*S. muticum*	Tetraprenyltoluquinol chromane meroterpenoid	[53]
*S. naozhouense*	Sargassumone (2R,6S,8S,9S)-hexahydro-2,9-dihydroxy-4,4,8-trimethyl-6-acetyloxy-3(2H)-benzofuranone (6S,8S,9R)-hexahydro-6,9-dihydroxy-4,4,8-trimethyl-2(2H)-benzofuranone Loliolide (+)-Epiloliolide Spheciospongones A (+)-Kjellmanianone	[54]
*S. sagamianum*	Chromequinolide 11′-Hydroxysargachromelide 15′-Hydroxysargaquinolide 15′- Methylenesargaquinolide (2′E,5′E)-2-Methyl-6-(7′-oxo-3′-methylocta-2′,5′-dienyl)-1,4-benzoquinone	[55]
*S. serrifolium*	Sargahydroquinoic acid Sargachromanol	[52,56,57]
	Sargahydroquinoic acid	[49]
	Sargaquinoic acid	[56]
*S. siliquastrum*	Sargahydroquinoic acid Isonahocol (Isomers D1; D2) Nahocol (Isomers A; A1; D1; D2)	[58]
	Sargachromenol (Isomer D; E; K) 13-(3,4-dihydro-6-hydroxy-2,8-dimethy2H-1-benzopyran-2-yl)-2,6,10-trimethyl-trideca-(2E,6E)- diene-4,5,10-triol 9-(3,4-dihydro-6-hydroxy-2,8-dimethy-2H-1-benzopyran-2- yl)-2,6-dimethyl-(6E)-nonenoic acid	[59]
*S. thunbergii*	Thumbergol (Isomer A; B)	[60]
	Sargachromanol (Isomer E; D) Sargahydroquinoic acid	[61]
	Indole-6-Carboxaldehyde	[62,63]
*S. tortile*	δ-Tocotrienol, δ -Tocotrienol; 11′,12′ (+)-Epoxide	[40]
*S. wightii*	2(α)-hydroxy-(28,29)-frido-olean-12(13),21(22)-dien-20-propyl-21- hex-4′ (Z)-enoate; 2(α)-hydroxy-(28, 29)-frido-olean-12(13), 21(22)-dien-20-prop-2(E)-en-21-butanoate; 2α-hydroxy-8(17), 12 E, 14- labdatriene; 3β, 6β, 13α-tri hydroxy 8(17), 12E, 14-labdatriene	[64]

**Table 2:** Examples of phytosterols identified from *Sargassum* spp.

Species	Phytosterol	Reference
*S. elegans*	β-Sitosterol;Fucosterol	[66]
*S. fusiforme*	24(*S*)-Saringosterol; Ergosterol; Fucosterol; Cholesterol; β-Sitosterol	[67,68]
*S. horneri*	Fucosterol; Saringosterol	[65,69]
*S. lacerifolium*	β-Sitosterol	
*S. piluliferum*	Ergosterol; Fucosterol; Cholesterol;	[68]
*S. thunbergii*	Ergosterol; Fucosterol; Cholesterol;	[68]

## Data Availability

The data that support the findings of this study are available on request from the corresponding author.
